# Case Study of Retroperitoneal Fibrosis: Multisystem Impact and Treatment Regiments

**DOI:** 10.51894/001c.144640

**Published:** 2025-09-30

**Authors:** Evan Schumacher

**Affiliations:** 1 College of Osteopathic Medicine Michigan State University

## 120

### INTRODUCTION

Retroperitoneal Fibrosis (RPF) is an inflammatory disease producing extracellular matrix composed of type I collagen fibers. RPF can be categorized based on its pathogenesis into two subtypes. Primary (idiopathic) RPF, driven by immune dysregulation increasing the release of IgG4 B-cell stimulating cytokines IL-4, IL-5, and IL-13 and anti-inflammatory cytokines IL-10 and TGF-β which stimulate fibroblast activation. Alternatively, Secondary RPF, results from increased localized inflammatory response through release of damage-associated molecular patterns (DAMPs) recruiting macrophages which secrete IL-1β, TNF-α, and TGF-β, lymphocytes, and neutrophils that release reactive oxygen species and damage tissue.

### CASE DESCRIPTION

A 67-year-old male with a history of hydronephrosis post ureteral stents, Chronic Kidney Disease (CKD) stage 3, testicular hydrocele, Cerebrovascular Accident (CVA) on anticoagulation, 3.7 cm Abdominal Aortic Aneurysm (AAA) presented with left lower extremity deep vein thrombosis and subsegmental pulmonary emboli, requiring IV heparin, thrombectomy, and transition to oral Rivaroxaban. Retrospective CT imaging revealed soft tissue encasing the aorta, iliac arteries, and ureters, leading to bilateral hydroureteronephrosis. Biopsy findings were consistent with retroperitoneal fibrosis (benign spindle cell proliferation, IgG4:IgG ratio <40%). The patient was initiated on mycophenolate mofetil (1,000 mg twice daily) and a short-term prednisone course.

### DISCUSSION/CONCLUSION

Initial management of RPF is ureteral decompression with surgical percutaneous nephrostomy tubes, double-J ureteral stents, ureterolysis followed by omental wrapping of the ureters, as well as conservative medical management with glucocorticoids independent or in conjunction with surgical intervention.

In a study of 12 individuals with RPF (11 completed treatment), prednisolone was administered at 60 mg on alternate days for two months, tapered to 5 mg over the next two months, and maintained at 5 mg daily for two years, with 9 patients experiencing symptom relief and mass regression, 1 requiring ureterolysis due to persistent mass, and 1 needing a small steroid dose for recurrence.

Recent advances in management of RPF have discovered the involvement of mammalian target of rapamycin (mTOR) signaling pathway prompting the development of a therapy combining a gradual reduction of prednisone with a long-term, stable dosage of sirolimus, demonstrating 70% reduction in acute inflammation and 50% decrease in fibrous tissue bulk.

